# Combination of Interleukin-6, C-Reactive Protein and Procalcitonin Values as Predictive Index of Sepsis in Course of Fever Episode in Adult Haematological Patients: Observational and Statistical Study

**DOI:** 10.3390/jcm11226800

**Published:** 2022-11-17

**Authors:** Daniela Carcò, Paolo Castorina, Paola Guardo, Valeria Iachelli, Tecla Pace, Paola Scirè, Rosaria Stanco, Uros Markovic, Giulio Antonio Milone, Federica Galbo, Gaetano Moschetti, Emanuele Martorana

**Affiliations:** 1 Istituto Oncologico del Mediterraneo, 95029 Viagrande, Italy; 2 Istituto Nazionale Fisica Nucleare, 95125 Catania, Italy; 3 Faculty of Mathematics and Physics, Charles University, 121 16 Prague, Czech Republic; 4 Division of Hematology, Azienda Ospedaliero Universitaria Policlinico “G.Rodolico-San Marco”, 95123 Catania, Italy

**Keywords:** haematological patients, transplantation, serum biomarkers, composite biomarker index

## Abstract

Haematological patients represent a vulnerable population to opportunistic infections, mainly due to the disease itself and chemotherapy-induced neutropenia. The level of immune suppression strongly increases the importance of timely antibiotic treatment in order to prevent sepsis-related mortality. During the initial fever episode, serum biomarkers are usually used to estimate the probability of blood stream infection prior to the results of microbial diagnosis. A new serum biomarker combination study on a febrile haematological population, including C-reactive protein (CRP), interleukin-6 (IL−6) and procalcitonin (PCT), is proposed in order to improve their predictive accuracy. In our prospective study, CRP, IL−6 and PCT were evaluated in 34 immunosuppressed haematological patients immediately after the onset of 51 fever episodes, either during the course of standard chemotherapy or high-dose chemotherapy and autologous stem cell transplant. The fever episodes were divided into documented infections and fever alone. Receiver operating characteristic analysis (ROC) was performed for each biomarker and a combination of all three biomarkers (multiROC) to define a new predictive index. Significant differences were evidenced between the two groups (documented infection and no infection) for both PCT and IL−6 (*p* = 0.03 and *p* = 0.035, respectively), but none for CRP (*p* = 0.1). The composite parameter is more reliable than any single biomarker alone, with an area under the curve (AUC) of 79% and with high sensitivity and specificity. IL−6 gave the closest response compared to the composite index. Composite parameters of serum biomarkers could be used for an early diagnosis of infection at fever onset in haematological patients.

## 1. Introduction

Sepsis represents one of the most common causes of ICU hospitalisation with elevated mortality rates [1]. Haematological patients are an extremely vulnerable population to opportunistic infections, mainly due to the disease itself and chemotherapy-induced neutropenia. Thus, early identification, diagnosis and antibiotic treatment become of great importance in order to prevent sepsis-related mortality. C-reactive protein (CRP), interleukin-6 (IL−6) and procalcitonin (PCT) have been widely used to facilitate sepsis diagnosis, with limited results [2,3,4,5,6,7,8,9,10,11]. Heart rate variability has also been considered as an important index for the diagnosis and prognosis of infection [12,13,14,15,16].

More recently, the two previous methods have been combined [17] to provide a more reliable approach for infection diagnosis at an early stage. Further refinement of the measurements is, however, necessary due to regular clinical procedures (continuous electrocardiography use plus daily serum collections [17]). On the other hand, it could be useful to verify if the combination of serum biomarkers at fever onset, without other vital clinical information, could improve the evaluation of infection probability [2,3]. The diagnostic accuracy during the first fever episode of CRP, PCT and IL−6 has been largely used to predict the probability of blood stream infection prior to the results of microbial diagnosis for immunosuppressed haematological patients during the course of chemotherapy [2,3,4,5,6,7,8]. 

With this aim, CRP, PCT and IL−6 were evaluated during a total of 51 fever episodes at the Mediterranean Oncology Institute (IOM). The biomarkers under study showed good classification quality, as also reported by other studies.

However, a more detailed and careful analysis could be obtained by investigating combinations of the previous biomarkers. In fact, biomarker combinations have been applied to oncological and haematological patients (see the recent review [18]). For example, in a recent paper [19], a combination of CRP and the neutrophil-to-lymphocyte ratio has been proposed as a novel prognostic index in patients with bladder cancer after radical cystectomy. Biomarkers pairs have been used to predict and/or prognosticate sepsis, including myeloid cells-1 (sTREM-1) and PCT or sTREM-1 and IL−6 [20,21,22]. The diagnostic performance indicated by the bioscore that combined the intensity of CD64 expression on polymorphonuclear cells together with PCT and sTREM-1 has been discussed in [22]. Yang et al. [23] propose the use of a CRP, PCT and the sepsis-related organ failure (SOFA) score to predict sepsis. Important problems in using multi-biomarker combinations in clinical practice include the wide variation in the threshold between markers and also the difficulty in judging which marker is preferred for the various inflammation diseases. For these reasons, here, a new combined index (CIndex) was calculated using CRP, PCT and IL−6 after the normalisation of their values by the mean, which shows a better classification quality (AUC: 0.79, sensitivity: 73% and specificity: 77%).

## 2. Materials and Methods

In this prospective study, CRP, PCT and IL−6 were evaluated immediately after 51 fever episodes of 34 haematological adult patients (>18 years old), affected by Hodgkin lymphoma (HL) (2/34), non-Hodgkin lymphoma (17/34) and multiple myeloma (MM) (15/34). Evaluated patients underwent either standard or high-dose chemotherapy, followed by autologous stem cell transplant. Patients with a hospital stay of ≤2 days were excluded. Fever was defined as an axillary temperature of >38 °C for >1 h, with or without chills. In Table 1, the population was divided into 2 groups with 40 and 11 cases for negative and positive blood cultures, respectively, describing the serum biomarker mean and median values.

The laboratory tests performed at fever onset included a complete blood cell count, measurement of serum levels of creatinine and blood urea nitrogen, and measurement of electrolytes, hepatic transaminase enzymes, and total bilirubin. At least three sets of blood cultures were taken, with a set collected simultaneously from each lumen of an existing central venous catheter, and from a peripheral vein site; furthermore, stool and urine cultures were analysed from each patient.

PCT was measured using an enzyme-linked fluorescent assay with BioMeriux kits on the VIDAS immunoassay system, with an upper limit of the normal range for serum PCT of 2 mg/mL.

The immuno-rate format for CRP was based on an enzymatic heterogeneous, sandwich immunoassay format provided by VITROS 5600 (Ortho Clinical Diagnostics). The precision of the method was evaluated with quality control materials on the VITROS 5600 Integrated System, following NCCLS protocol EP5. The upper limit of the normal range for serum CRP was 1 ng/mL.

The IL−6 test was performed using the VITROS5600 system (Ortho Clinical Diagnostics) and a two-step immunometric technique. The precision of the method was estimated with quality control materials on VITROS 5600. The upper limit of the normal range for serum IL−6 was 6.65 pg/mL.

In addition to the value of PCT, CRP and IL−6, we developed a new index called the combined index (CIndex), which was formulated as follows:

(1) $$CIndex_{i}=\frac{PCT_{i}}{\hat{PCT}}+\frac{CRP_{i}}{\hat{CRP}}+\frac{IL6_{i}}{\hat{IL6}}$$

where i indicates the i−th fever onset with relative laboratory tests and $\hat{PCT}$, $\hat{CRP}$ and $\hat{IL6}$ represent the mean values of the entire sample. The normalisation of the values to the mean is a crucial step to avoid different threshold scales. For example, if the value of each biomarker was equal to its average, then the CIndex = 3. On the other hand, some biomarkers are more reliable than others at predicting sepsis at the onset of fever and they have different, effective, prognostic weights in the CIndex and in the receiver operating characteristic (ROC) analysis, although a linear combination without coefficients is used.

Regarding the statistical analysis, a *p*-value less than 0.05 was considered statistically significant, while data comparison was carried out using the non-parametric Wilcoxon–Mann–Whitney test. ROC curves were used to show the specificity and sensitivity for the cut-off points of each serum biomarker value, in order to maximise both specificity and sensitivity. The statistical analysis was initially carried out for each single biomarker and then for the combined index with a multi-ROC study, by using R [24] and RStudio [25] with the following packages: ROCR [26] and multiROC [27] for the analysis of ROC curves; ggridges [28] for density probability; introdataviz [29] for violin plot and ggplot2 [30] for data visualisation.

## 3. Results

**Figure 1:**
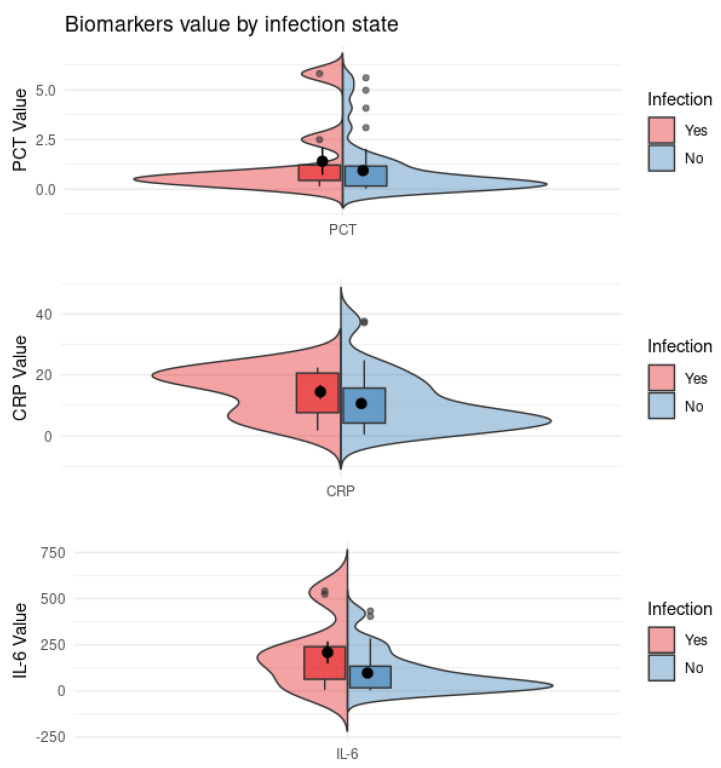
The plot presents the distribution for the three biomarkers under different conditions for both group 1 and group 2. **Top**: PCT value distribution; **middle**: CRP value distribution; **bottom**: IL−6 value distribution.

**Figure 2:**
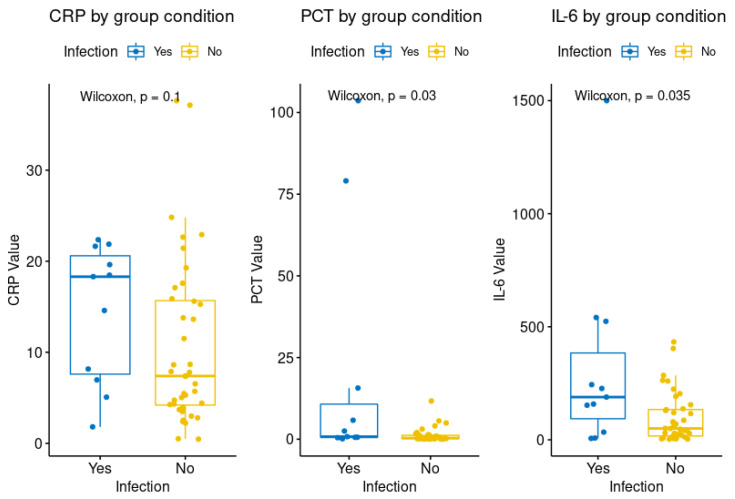
Boxplot and statistical significance of biomarkers for group 1 (no infection) and group 2 (infection).

A total of 51 onset first fever episodes were studied, including 40 cases (group 1) with at least 1 peak in body temperature of >38 °C in the absence of laboratory evidence of infection and 11 cases (group 2) with microbiologically documented infection (positive blood). The case and control group showed some interesting differences in their distributions for all three diagnostic parameters (CRP, PCT and IL−6). Figure 1 represents a split violin diagram that compares data distributions based on infection conditions. As shown in Figure 2, there were significant differences between the two groups for both PCT and IL−6 (*p* = 0.03 and *p* = 0.035, respectively), but none for CRP (*p* = 0.1).

**Figure 3:**
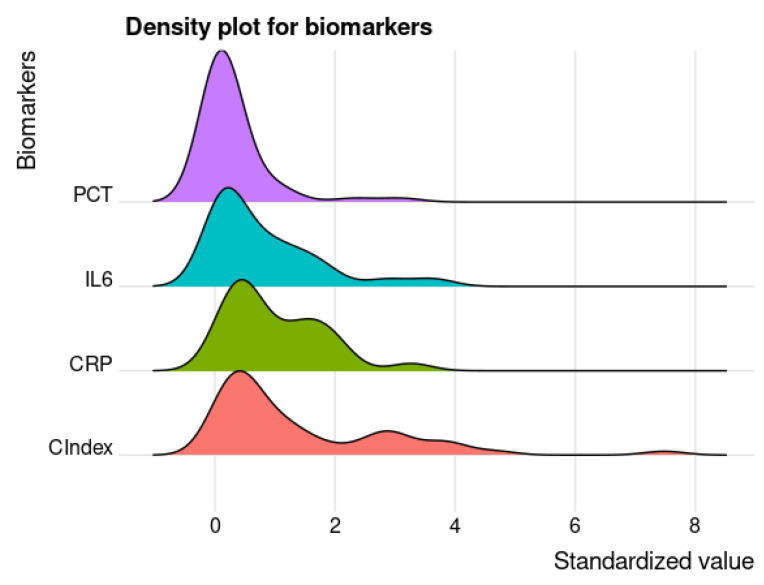
Density plot for PCT, IL−6, CRP and CIndex. *X*-axis shows the value for each biomarker normalised by the mean value, while the *Y*-axis represents the density probability.

In Figure 3, the density probability functions for all serological inflammatory biomarkers with the CIndex score are presented and the double hump in the CIndex curve highlights the binary classification with the threshold value.

**Figure 4:**
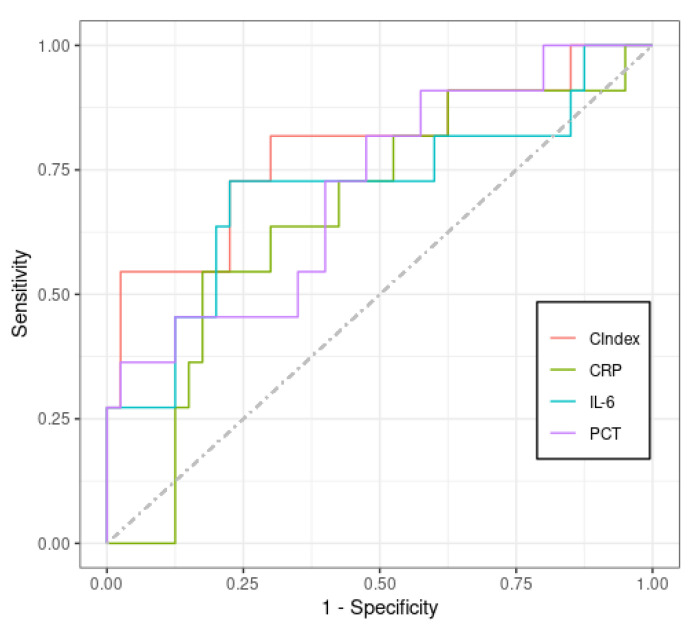
Comparison of ROC curves, including the new CIndex that demonstrates better classification than the others.

The ROC analysis, as reported in Figure 4, showed that all the biomarkers are good classifiers, and the AUC, cut-off, sensitivity and specificity values for PCT, CRP, IL−6 and the CIndex are given in Table 2. The combined index (CIndex) was calculated using CRP, PCT and IL−6 after the normalisation of their values by the mean and shows better classification quality (AUC: 0.79, sensitivity: 73% and specificity: 77%). Despite all biomarkers having good predictive accuracy, the CIndex demonstrated a better AUC, which indicates better classification in the study population between the two groups, although sensitivity and specificity are the same when using IL−6 alone.

## 4. Discussion

The role of biomarkers in the diagnosis and prognosis of sepsis is well known [2,3,4,5,6,7,8,9,10,11,12,13,14,15,16,17,18,19,20,21,22,23,31]. However, to date, no standardised use of individual or a combination of biomarkers has been established for the early detection of infection.

We analysed a single-centre haematological population at IOM between 2021 and 2022 with the purpose of exploring a potential combined use of the known serum biomarkers, thus improving the prediction of infectious episodes, after fever onset.

A new, dimensionless, easy to use index (CIndex) has been proposed. By definition (see Equation (1)), it reduces the effect of measurement fluctuations and normalises each laboratory test value to its mean.

It is useful to clarify the following aspect. The cut-off values of the single biomarkers are reported in Table 2 and a meaningful combined index is difficult to define without the correct normalisation of these values to their mean values. Therefore, for clinical application, an evaluation of the mean values is required, which can be obtained by large data sets (if available) or after the collection of a sample of patients with homogeneous conditions (e.g., environment; social security system), as is the case in our study.

The comparison of the statistical predictivity among the CIndex, PCT, CPR and IL−6 in the early diagnosis of sepsis showed that a large AUC with high sensitivity and specificity can be obtained by the proposed combination of the usual biomarkers. Table 2 clearly shows that IL−6 has a larger statistical weight in the CIndex than the other biomarkers. In this respect, IL−6 “drives” the CIndex, but the other inflammatory parameters contribute to increasing the AUC by about 10%, with respect to the single IL−6 case. The combination of three sentinel biomarkers is uncommon in clinical practice and we are aware that further analyses are needed in order to verify the CIndex as a potential reliable tool in early sepsis prediction. However, the new proposed index is quantitative, indicating a precise cut-off value of the combination of biomarkers, and is simple to apply. In this respect, it is useful to compare the predictive power of the CIndex with the combination of CRP, PCT and SOFA [23]. The A.U.C obtained by the CIndex and that obtained in ref. [23] are equal (0.79). The difference is mainly in the IL−6 values (A.U.C. of 0.77 in our sample) in comparison with SOFA (0.67). However, the most important difference between the two methods is that for the CIndex, the normalisation was carried out using the average values of the biomarkers, giving a continuum index, whereas the combination of CRP, PCT and SOFA requires each measured clinical variable (using a rounded number) to be converted into categorical values as a final weighted value (see additional file 1: Table S3, Ref. [23]). Moreover, it should be stressed that our approach applies to sepsis diagnosis immediately after the first fever episode for haematological patients, which is an important specific point of our analysis. Indeed, a more stringent comparison could be required with the biomarker combinations for haematological patients in ref. [31], in which different methods have been applied. Indeed, in [31], the combination of two biomarkers (CRP+PCT, or PCT+presepin) was analysed, but CRP was assessed daily during the whole neutropenia period, while PCT or presepsin were measured during the first 48 h after the onset of febrile episodes. This is in contrast to our study, where all the biomarkers were measured immediately after the onset of neutropenia (less than 12 h). From a statistical point of view, one could expect that an increase in the number of biomarkers involved in the combination should trivially improve the predictive power. First, this could not be the case, since it depends on the reliability of the single biomarker in predicting sepsis at the onset of fever; moreover, the application of six or more inflammatory parameters is clinically and statistically difficult to handle.

## Figures and Tables

**Table 1:** A summary statistic of the entire sample and group conditions for the biomarkers. The table shows, from left to right, the following information: “Group”: the group to which the measure belongs (“Total” refers to the entire sample; “Sepsis” refers to those with fever episodes with corresponding documented infections (positive blood, urine or stool culture) and “No sepsis” means without documented infection); “N”: cardinality of the group; “Mean”: the arithmetic mean; “Median”: the arithmetic median; “SD”: standard deviation; “Q1”: first quartile and “Q3”: third quartile.

Biomarker	Group	N	Mean	Median	SD	Q1	Q3
CRP (ng/mL)	Total	51	11.44	7.90	8.94	4.35	17.95
	Sepsis	11	14.45	18.30	7.55	7.60	20.60
	No sepsis	40	10.62	7.40	9.19	4.20	15.68
PCT (mg/mL)	Total	51	5.05	0.50	18.01	0.20	1.44
	Sepsis	11	19.05	0.79	36.45	0.53	10.73
	No sepsis	40	1.21	0.37	2.15	0.16	1.22
IL−6 (pg/mL)	Total	51	146.35	69.40	235.25	19.40	190.50
	Sepsis	11	325.81	189.00	0.04	93.45	384.00
	No sepsis	40	97.00	50.20	109.72	16.98	134.00

**Table 2:** Values for ROC curve performance measurements for a chosen cut-off value, maximising both sensitivity and specificity.

Biomarker	AUC	Cut-Off	Sensitivity	Specificity
PCT	0.72	0.59	60%	73%
CRP	0.66	14.6	64%	70%
IL−6	0.71	153	73%	77%
CIndex	0.79	1.72	73%	77%

## Data Availability

Not applicable.
